# Chromosomal Inversions Mediated by Tandem Insertions of Transposable Elements

**DOI:** 10.1093/gbe/evaf131

**Published:** 2025-08-21

**Authors:** Robin Aasegg Araya, William B Reinar, Ole K Tørresen, Clément Goubert, Tara J Daughton, Siv Nam Khang Hoff, Helle Tessand Baalsrud, Marine Servane Ono Brieuc, Anna Zofia Komisarczuk, Sissel Jentoft, José Cerca, Kjetill S Jakobsen

**Affiliations:** Centre for Ecological and Evolutionary Synthesis (CEES), Department of Biosciences, University of Oslo, Oslo 0316, Norway; Centre for Ecological and Evolutionary Synthesis (CEES), Department of Biosciences, University of Oslo, Oslo 0316, Norway; Section for Genetics and Evolutionary Biology, Department of Biosciences, University of Oslo, Oslo 0316, Norway; Centre for Ecological and Evolutionary Synthesis (CEES), Department of Biosciences, University of Oslo, Oslo 0316, Norway; R. Ken Coit College of Pharmacy, University of Arizona, Tucson, AZ 85721, USA; Centre for Ecological and Evolutionary Synthesis (CEES), Department of Biosciences, University of Oslo, Oslo 0316, Norway; Centre for Ecological and Evolutionary Synthesis (CEES), Department of Biosciences, University of Oslo, Oslo 0316, Norway; Centre for Ecological and Evolutionary Synthesis (CEES), Department of Biosciences, University of Oslo, Oslo 0316, Norway; Department of Animal and Aquacultural Sciences, Faculty of Biosciences, Norwegian University of Life Sciences, Ås NO-1433, Norway; Centre for Ecological and Evolutionary Synthesis (CEES), Department of Biosciences, University of Oslo, Oslo 0316, Norway; Centre for Ecological and Evolutionary Synthesis (CEES), Department of Biosciences, University of Oslo, Oslo 0316, Norway; Centre for Ecological and Evolutionary Synthesis (CEES), Department of Biosciences, University of Oslo, Oslo 0316, Norway; Centre for Ecological and Evolutionary Synthesis (CEES), Department of Biosciences, University of Oslo, Oslo 0316, Norway; Department of Bioinformatics and Genetics, Swedish Museum of Natural History, Stockholm 114 18, Sweden; Centre for Ecological and Evolutionary Synthesis (CEES), Department of Biosciences, University of Oslo, Oslo 0316, Norway

**Keywords:** Atlantic cod, chromosomal inversions, ectopic recombination, *hAT* transposons, MITEs, transposable elements

## Abstract

Chromosomal inversions play a crucial role in evolution by influencing phenotypes through the linkage of coadapted alleles. While inversions have been found across a large number of taxa, mapping and characterizing inversion breakpoint regions remain challenging, often due to the presence of complex tandem repeats and transposable elements. Here, we identify and quantify transposable elements in the breakpoints of the four large-scale inversions previously reported in Atlantic cod, leveraging on three high-quality long-read-based reference genome assemblies for the Norwegian Coastal cod, the Northeast Arctic cod, and Celtic cod ecotypes. We detected a significant enrichment of transposable element orders and superfamilies with terminal inverted repeats within the inversion breakpoint regions of chromosomes 1, 7 and 12. Notably, we discovered a tandem accumulation of miniature inverted-repeat transposable elements belonging to a family of *hAT* transposons, exclusively residing in the breakpoints of the inverted haplotype on chromosomes 1 and 7 found in the Northeast Arctic cod. The accumulation of tandemly arranged transposable elements with high sequence similarity in breakpoint regions suggests that they have driven the appearance of inversions through ectopic recombination, further supporting the potential of transposable elements in facilitating chromosomal reorganizations with large evolutionary implications.

Significance Atlantic cod populations and ecotypes can be discriminated by chromosomal inversions that influence phenotypes through linkage of coadapted alleles. Here, we have investigated four large inversions in the Atlantic cod and found that the breakpoints are enriched in transposable elements. The repeat structures caused by these transposable elements are complex, including tandemly arranged inverted repeats, and they tend to only reside in the breakpoints of the inverted haplotype while being absent in the noninverted haplotype. The accumulation of long tandem insertions of similar transposable elements in breakpoints suggests that they have driven the evolution of these inversions.

## Introduction

Genetic variation is essential for evolution and can arise from various sources, including single-point mutations, transposable element (TE) activity, and structural variants like chromosomal inversions (Sætre and Ravinet 2019). In particular, inversions have been shown to include sets of coadapted allelic variants within the regions they encompass (Wellenreuther and Bernatchez 2018; Mérot et al. 2020; Gutiérrez-Valencia et al. 2021; Villoutreix et al. 2021) that can potentially have an important effect on the fitness of the individuals. As a result, inversions have important evolutionary implications for the processes of adaptation (Faria et al. 2019; Mérot et al. 2020) and speciation (Wellenreuther and Bernatchez 2018). Examples of the impact of inversions include variation in mimicry patterns associated with local adaptation in the butterfly species *Heliconius numata* (Jay et al. 2018), different life history strategies related to mating in ruffs (*Calidris pugnax*) (Lamichhaney et al. 2016), and migratory spawning behavior in Atlantic cod (*Gadus morhua*) (Berg et al. 2016).

**Fig. 1.**
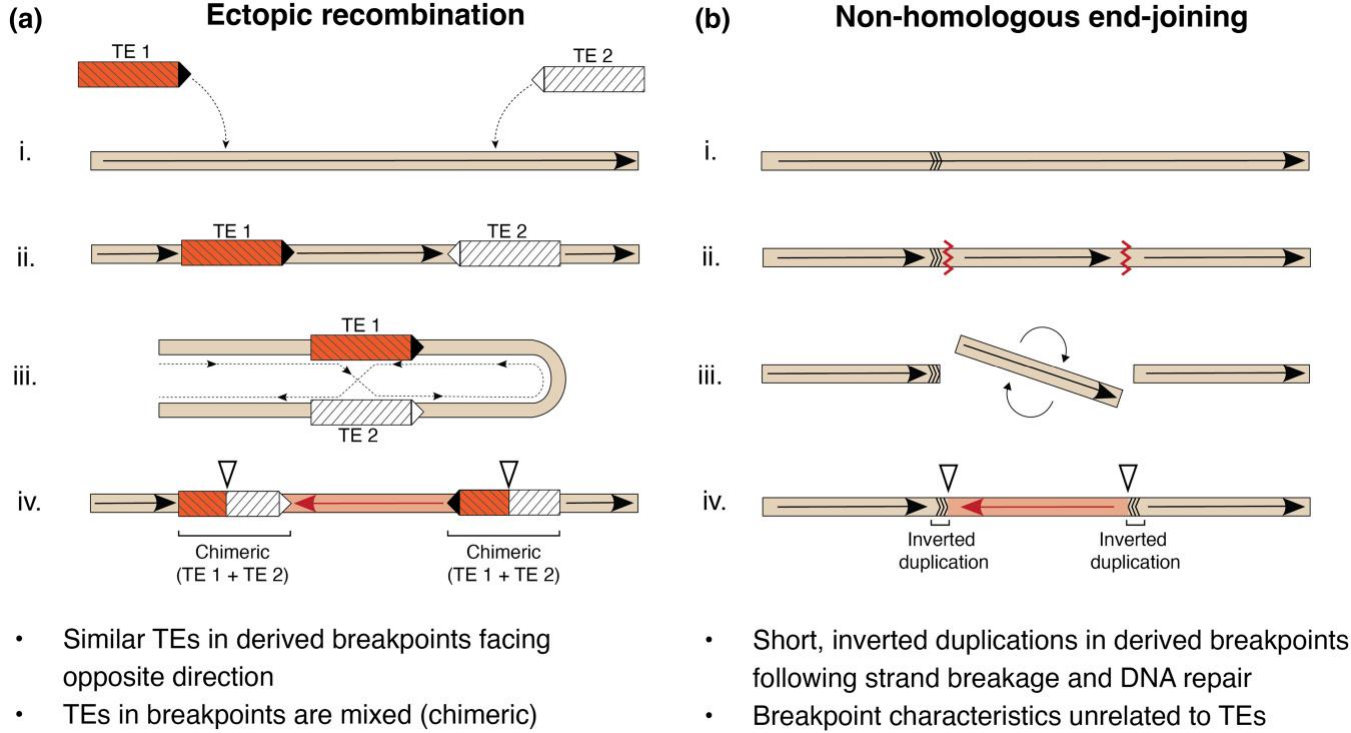
Schematic representation of molecular mechanisms leading to an inversion. a) An ectopic recombination scenario involving (i) insertions of two TE copies of the same family (high sequence similarity) in (ii) opposite directions. (iii) Misalignment and recombination between TE copies leads to (iv) an inversion, resulting in a signature of two chimeric TEs, which overlap the breakpoints (shown as open triangles). Red (TE1) and white (TE2) coloration indicates different TE copies, and arrowheads show the direction of the TE insertion. b) (i) A NHEJ scenario involving (ii) strand breakage in two locations (red zigzagged lines), (iii) flipping of the mid-segment, and ligation of the ends leads to an inversion flanked by inverted duplications (shown as arrow triplets—initially only present in the proximal location before strand break). See Furuta et al. (2011) for an in-depth description of the molecular mechanisms leading to NHEJ. The genomic signatures left in the derived breakpoints are listed as bullet points below each scenario.

Despite the growing appreciation of the evolutionary impact of chromosomal inversions, the molecular mechanisms by which inversions originate are yet to be fully explored in a genomic context. Double-stranded or single-stranded staggered breaks on the same chromosome repaired by nonhomologous end joining (NHEJ) can result in an inversion (Ranz et al. 2007; Gu et al. 2008; Furuta et al. 2011; Guillén and Ruiz 2012; Burssed et al. 2022). Inversions can also result from ectopic recombination (Kupiec and Petes 1988; Lim and Simmons 1994; Gu et al. 2008; Ling and Cordaux 2010; Guillén and Ruiz 2012; Burssed et al. 2022), in which recombination happens between similar sequences that are present in different genomic regions, such as homologous TEs in opposite directions on a chromosome (Fig. 1). Identifying the underlying mechanisms responsible for the generation of inversions, however, can be challenging (Faria et al. 2019), and the relative contributions of NHEJ and ectopic recombination have not been investigated in detail, with few exceptions, such as the breakpoint comparisons of almost 30 different inversions in fruit flies (Ranz et al. 2007; Guillén and Ruiz 2012), as well as the presence of long inverted repeats spanning the breakpoints of major inversions in mammals (Harringmeyer and Hoekstra 2022) and in fish (Meyer et al. 2024). The advent of high-quality chromosome-level genomes (Peona et al. 2018; The Darwin Tree of Life Project Consortium et al. 2022) and new bioinformatic tools provides promising avenues for understanding the mechanisms that generate inversions.

The Atlantic cod (*G. morhua*) genome enharbors four large chromosomal inversions that have been linked to adaptations to environmental and ecological conditions such as temperature, light, oxygen, and salinity at different geographical sites (Berg et al. 2015, 2017; Barth et al. 2017, 2019) and distinguish the two ecotypes: the migratory North East Arctic cod (NEAC) and the stationary Norwegian coastal cod (NCC) (Berg et al. 2016; Matschiner et al. 2022). In a recent study, Matschiner et al. (2022) demonstrated that the migratory ecotype (NEAC) is associated with the derived (inverted) haplotype of the inversions on chromosomes 1 and 7, while the ancestral (noninverted) haplotype is predominant in the nonmigratory ecotype (NCC) (Matschiner et al. 2022). Conversely, the nonmigratory NCC is associated with the derived (inverted) haplotypes on chromosomes 2 and 12, whereas the ancestral (noninverted) arrangements are mostly found in the migratory ecotype (Matschiner et al. 2022). The four inversions originated at different ages from 0.40 to 1.66 million years ago (Matschiner et al. 2022), suggesting that they evolved within the Atlantic cod lineage.

The presence of these four large chromosomal inversions offers a unique opportunity to study their evolutionary origin. Specifically, it is possible to investigate if the different inversions have originated from similar molecular processes or not. One way to investigate this is to look at the inversion breakpoints. If ectopic recombination is involved, we expect to find an accumulation of similar sequences—such as homologous parts of related TEs—in the inversion breakpoint regions, as shown in previous studies (Cáceres et al. 1999; Zhang and Peterson 2004; reviewed in Böhne et al. 2008; Delprat et al. 2009; Guillén and Ruiz 2012; Sarilar et al. 2014; Sharma and Peterson 2023). In such cases, TEs involved in causing the inversion can be detected as chimeras following the recombination event (Fig. 1), which can lead to aggregations of similar TEs in the breakpoint pairs. Although TE accumulations may also result from suppressed recombination in the breakpoints, such accumulations are not necessarily homologous and do not have a causal link to the inversion formation. Conversely, if NHEJ is involved in forming the inversion, we expect to observe short, inverted duplications of nonrepetitive DNA in the breakpoints, unrelated to TE insertions, representing the signature left by strand transfer and ligation during DNA repair (Fig. 1) (Furuta et al. 2011; Guillén and Ruiz 2012). However, characterization of the breakpoint regions in Atlantic cod at the sequence level has not been done. Doing so will elucidate the underlying mechanisms and origins of inversions, which have often been overlooked (reviewed in Villoutreix et al. 2021).

Here, we perform a survey of TEs associated with the four chromosomal inversions on chromosomes 1, 2, 7, and 12 in Atlantic cod. Using synteny analyses and PacBio HiFi sequencing, we precisely locate inversion breakpoints and manually curate the TEs in breakpoint pairs for the NEAC and NCC ecotypes. We identify accumulations of specific TE families overlapping the breakpoints of all four inversions, with enrichments of certain TEs containing terminal inverted repeats (TIRs). We find tandemly arranged miniature inverted-repeat TEs (MITEs) related to the DNA transposon *hAT* superfamily in the breakpoints on chromosomes 1 and 7. Additional population-level analyses of the chromosome 2 inversion in 58 cod individuals reveal conserved breakpoint locations between individuals, also in terms of TE insertions. Overall, our findings suggest that TEs are directly involved in recombination events leading to inversion formation and thereby indirectly contribute to local adaptation.

## Results

### Inversion Breakpoints Are Associated with Accumulations of Specific TE Superfamilies

To determine the genomic coordinates of the inversions on chromosomes 1, 2, 7, and 12, we used a combination of chromosome alignments and mapping of raw reads (see Materials and Methods; Table S1 and Figs. S1 to S4) onto the NEAC, NCC, and Celtic cod reference genome assemblies. This led us to identify each of the different breakpoint intervals to the scale of a few thousand base pairs (mean breakpoint interval size: 16,862 bp; SD: 21,417 bp; Table 1).

**Table 1:** Overview of breakpoint coordinates in the Atlantic cod inversions

	Breakpoint coordinates			Arrangement	
Chr	A	B	C	Derived	Ancestral
1^a^	11,296,105-11,302,315	19,180,947-19,187,825	28,333,982-28,336,075	NEAC	NCC
2	21,848,272-21,858,616	26,151,340-26,163,504	…	NCC	NEAC
7	16,838,295-16,871,460	26,338,531-26,410,445	…	NEAC	NCC
12	638,394-646,752	13,659,852-13,660,483	…	NCC	NEAC

All coordinates refer to the assembly carrying the derived (inverted) haplotype. The coordinates were determined by split read alignment and are provided as ranges.

^a^The inversion on chromosome 1 is a double inversion with adjacent breakpoints (B).

Following identification of inversion breakpoints, we created a species-specific, manually curated TE library using an in-house pipeline (see Materials and Methods; Table S2 and Fig. S5). The resulting library masked 35.23% of the NEAC assembly, with 28.3% being interspersed repeats (Table S3), and showed an even distribution of DNA TEs, long terminal repeat (LTR) retrotransposons, and long interspersed nuclear elements (LINEs) with on average low divergence from their consensus sequences (mean divergence: 11.33%; SD: 11.65; see Fig. S6). Similar results were observed when masking the NCC and Celtic cod assemblies, indicating no substantial differences in TE annotations across the three cod ecotypes (Fig. S6). The TE annotations exhibited an NTE50 of 86,886 TEs (i.e. number of TE insertions required to annotate 50% of the genomic repeats) and LTE50 of 465 bp (i.e. the minimum length of annotated TEs needed to cover 50% of the genomic repeats), which is an improvement from the initial uncurated library, with shorter and more fragmented annotations (NTE50 = 123,454 TEs and LTE50 = 344 bp).

**Fig. 2.**
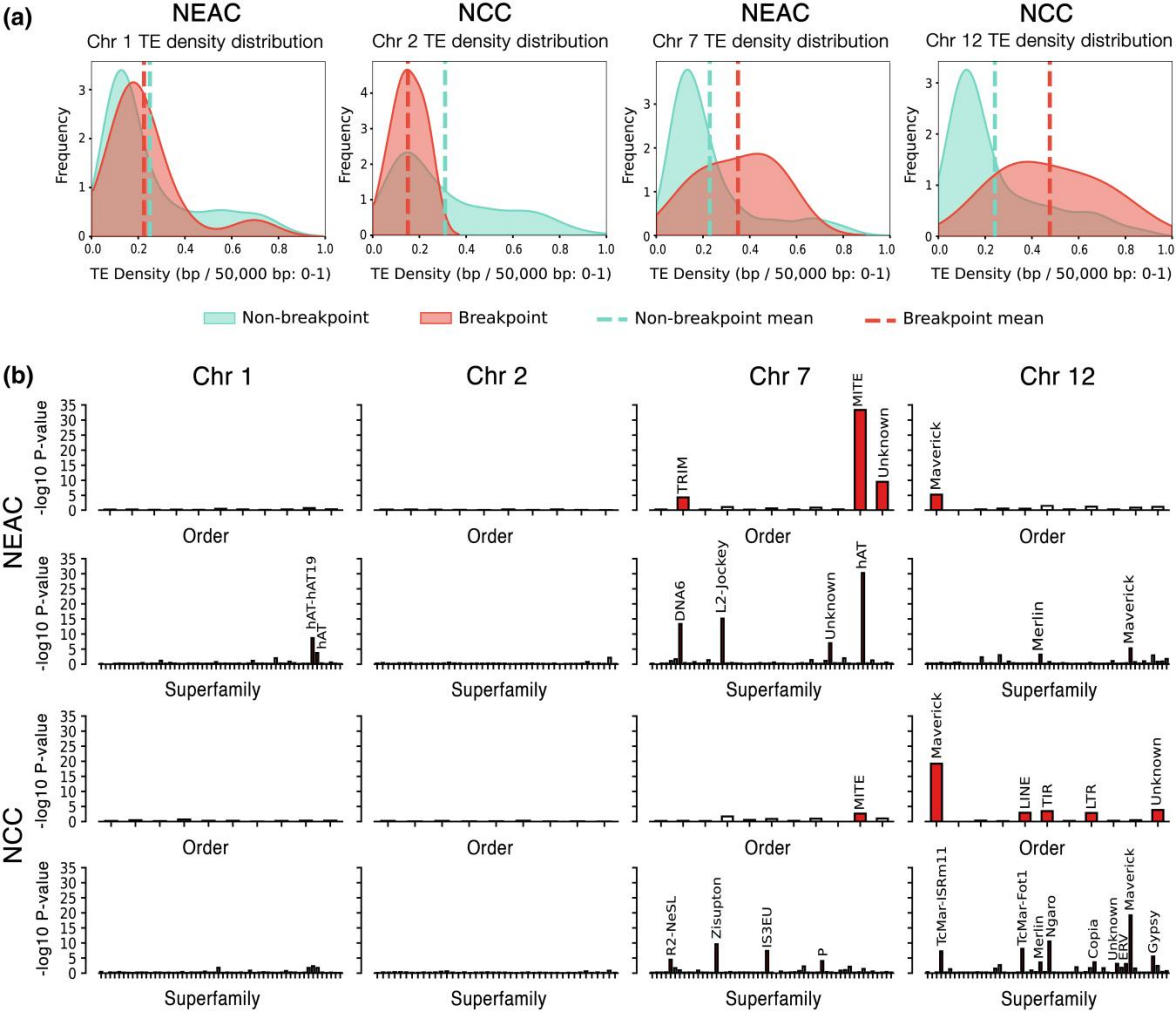
TE enrichment in breakpoints versus nonbreakpoint regions. a) Density distribution of TEs across breakpoint and nonbreakpoint regions on chromosomes 1, 2, 7, and 12, using 50-kb nonoverlapping sliding windows. The red curve represents TE density within breakpoint regions, while density in nonbreakpoint regions is shown in blue. Mean densities for breakpoint and nonbreakpoint regions are indicated by red and blue dotted lines, respectively. b) TE orders and superfamilies associated with breakpoint regions using a Student's *t*-test. TE orders and superfamilies are shown on the *x*-axis and −log_10_  *P*-values on the *y*-axis for chromosomes 1, 2, 7, and 12 in NEAC and NCC. Red bars indicate TE orders and superfamilies with significantly elevated densities within breakpoint regions.

To investigate the relationship between TEs and inversion breakpoints, we used our TE library to assess TE enrichment around the breakpoints on chromosomes 1, 2, 7, and 12, comparing these regions to the overall chromosomal TE densities. To capture the full TE content surrounding each breakpoint, we defined extended breakpoint regions of the inversions as the midpoint of each estimated breakpoint from Table 1 and extended it by 50 kb in both directions to include flanking sequences, resulting in breakpoint regions of the same 100-kb size (see Materials and Methods). We observed significantly elevated TE densities in the inversion breakpoints on chromosomes 7 and 12 of the derived haplotypes, compared to nonbreakpoint regions of 50 kb on the same chromosomes (Fig. 2a; Table S4) (Student's *t*-test with *P*-values: 5.42 × 10^−5^ and 6.4 × 10^−6^, respectively, excluding putative centromeric regions as estimated in Table S5 and Figs. S7 to S9). In addition, the syntenic breakpoint regions of the ancestral (noninverted) haplotype on chromosome 12 exhibited higher TE densities than the chromosomal average (*P*-value: 0.025). Chromosomes 1 and 2 did not display the same increase in general TE density around the breakpoints (Fig. 2a; Fig. S10a and d).

Further analysis of TE orders revealed above-average accumulations of specific TE orders within certain inversion breakpoints, where mean densities in the breakpoints exceeded 2 SD above the chromosome-specific mean (excluding putative centromeric regions; Fig. S10e and f). In particular, we identified high densities of MITEs in the breakpoints of the derived (inverted) haplotype on chromosome 7 (breakpoint mean density: 6.5%; SD: 1.3% vs. chromosomal mean density: 0.4%; SD: 1.0%). Additionally, visual inspection of individual breakpoints showed an enrichment of class II DNA elements (density peaks above 2 SD of chromosomal mean) in the breakpoints of the derived (inverted) haplotypes on chromosomes 1 and 7, specifically of TIRs and MITEs (Fig. S10e). We also observed elevated densities of LTR retrotransposons within two of the derived breakpoints on chromosome 1. On chromosome 12, we found elevated levels of TIRs, retroelements, and Maverick elements in at least one of the derived breakpoints and ancestral syntenic breakpoint regions (Fig. S10e and f).

To better quantify the relationship between TE orders and inversion breakpoints, we performed statistical testing of TE order density differences between breakpoint and nonbreakpoint regions within 50-kb sliding windows, using a Student's *t*-test. This revealed a significant tendency for MITEs to accumulate in the breakpoints of the chromosome 7 inversion (but not in the ancestral, syntenic breakpoint regions) (Fig. 2b). Similarly, the breakpoints of the derived chromosome 12 inversion showed significantly elevated densities of Maverick elements, but to a lesser extent in the syntenic regions of the ancestral haplotype (Fig. 2b).

Finally, we extended this analysis to the TE superfamily level, performing the same density comparison within 50-kb sliding windows for breakpoints versus nonbreakpoint regions using a Student's *t*-test. This yielded significant signals of enrichment for certain superfamilies of TIR elements (*hAT* elements, DNA-6, and unspecified MITEs) in the breakpoints of the derived chromosome 7 inversion, along with a superfamily of LINEs (Fig. 2b; Fig. S10e). For the chromosome 12 inversion, we again detected significantly elevated densities of an unspecified superfamily of Maverick elements within the derived breakpoints (Fig. 2b; Fig. S10e and f).

### Closely Related TEs Occupy Both Breakpoints of All Four Inversions in Atlantic Cod

**Fig. 3.**
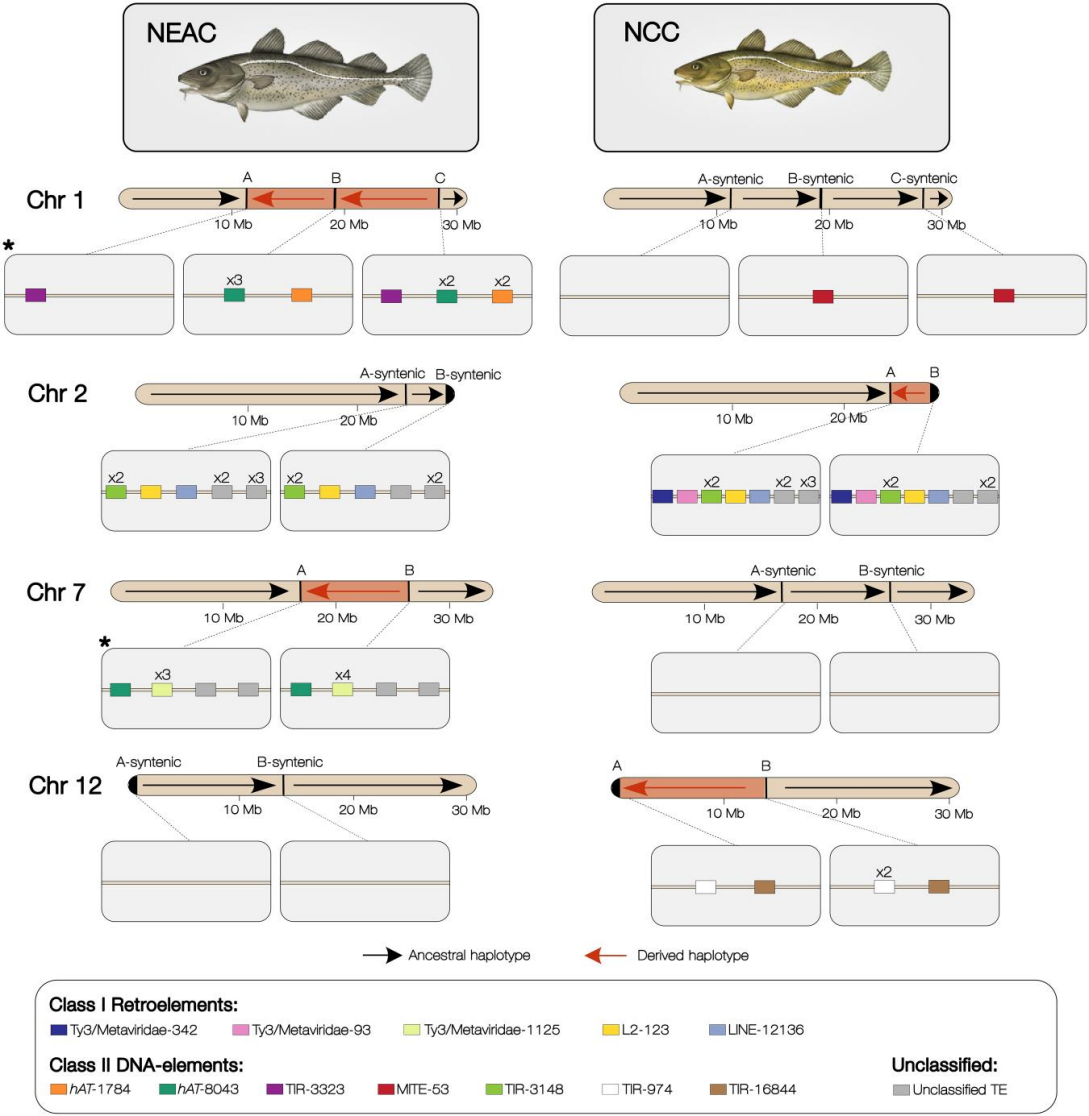
TEs in the inversion breakpoints of Atlantic cod. TE families present in both breakpoints of an inversion in chromosomes 1, 2, 7, and 12 in NEAC and NCC are displayed as squares, with colors corresponding to TE families as indicated in the legend. Each chromosome shows breakpoint and syntenic breakpoint regions (midpoint ± 50 kb) within the light-gray boxes below. Black arrows (rightward) on the chromosome cartoons show the ancestral (noninverted) haplotype, and red arrows (leftward) show the derived (inverted) haplotype. Insertion lengths and orientations of the TE families are not shown. Multiple insertions are indicated with an x and number of copies. Chromosomes are drawn to scale, with sizes indicated in Mb. * indicates breakpoints with a significantly higher occurrence of long TE insertions (>1,000 bp) of the same family, compared to randomly selected nonbreakpoint regions (see Fig. S13).

Given the apparent association between inversion breakpoints and specific types of TEs, we wanted to investigate the sequence similarity of family-specific TE accumulations between breakpoint pairs (i.e. breakpoints of the same inversion). Using the same 50-kb breakpoint regions as described above, we identified multiple similar sequence fragments between breakpoints belonging to the same inversion by dot plotting each breakpoint pair (sequence identity ≥ 25%) (Fig. S11). For all four derived (inverted) haplotypes, these fragments contained TE copies that were classified into the same family (Fig. 3), explaining the sequence similarity between the breakpoints. On chromosome 1, we found three different TE families from the class II DNA elements that were present in the three breakpoints of the derived haplotype (i.e. the derived double inversion), whereas these TEs were absent from the ancestral syntenic regions (where we found short insertions of another family of DNA elements in two of the syntenic “breakpoints”) (Fig. 3). A similar pattern was observed for the chromosome 12 inversion, where two families of class II DNA elements were found only in the inverted (derived) haplotype and not in the noninverted (ancestral) haplotype (Fig. 3). On chromosome 2, both inversion haplotypes showed a diversity of accumulated retroelements, primarily from the LTR retrotransposons (Fig. 3). Chromosome 7 exhibited insertions of various putative TE families (both LTR retrotransposons and DNA elements) in the breakpoints of the derived (inverted) haplotype, while two families of retroelements were present only in the syntenic breakpoint regions of the ancestral haplotype (Fig. 3). Manual inspection of consensus sequences and insertions confirmed that the breakpoint insertions consistently represented the same or overlapping portions of their respective TE family consensus sequence (Table S6).

The TE families that were present in breakpoint pairs (Fig. 3) varied from 2 to 8 copies and exhibited diverse consensus lengths, ranging from approximately 284 to 9,034 bp (Table S6). These TE families were not exclusively found in breakpoints (Fig. S12). Due to the accumulation of TEs from the same family in breakpoints, we compared the probability of finding such an accumulation of family-specific TEs in the breakpoint pairs versus random genomic regions. Specifically, we compared counts of observed family-specific TEs within both breakpoints of each inversion, to the average observed family-specific TEs occurring in 1,000 randomly selected region pairs on the same chromosome (Fig. S13). This showed that the chance of finding long TEs (i.e. >1,000 bp) of the same family in two randomly drawn genomic regions is low—for instance, within 1,000 randomly distributed region pairs of 100 kb in chromosomes 1 and 7—we expect to find on average less than 0.2 TEs of the same family (Fig. S13a). Conversely, we find insertions of specific TE families (Fig. 3) longer than 1,000 bp in all of the breakpoints on chromosomes 1 and 7 in the derived haplotypes, strengthening the association between TEs and these inversion breakpoints. When considering short family-specific TEs (i.e. below 1,000 bp), we did not observe any deviation from the average TE count in random regions (Fig. S13).

Although the association between inversion breakpoints and particular TEs suggests a role for TEs in facilitating inversion formation by ectopic recombination, we also evaluated whether other molecular mechanisms (i.e. NHEJ) could be involved in forming the inversions (see Fig. 1). By assessing structural variants in the inversion breakpoint regions (see Materials and Methods), we identified a 910-bp non-TE-derived inverted duplication nearby breakpoints B and C of the double inversion on chromosome 1 (Fig. S14). The inverted duplication in breakpoint C was located ∼23-kb upstream of the breakpoint boundary (Table 1) and thus evaluated as less likely to be involved in the inversion formation than the class II DNA elements present in the same breakpoints.

### Accumulation of *hAT* transposons in Chromosome 1 and 7 Breakpoints: Evidence for Tandem MITE Insertions

**Fig. 4.**
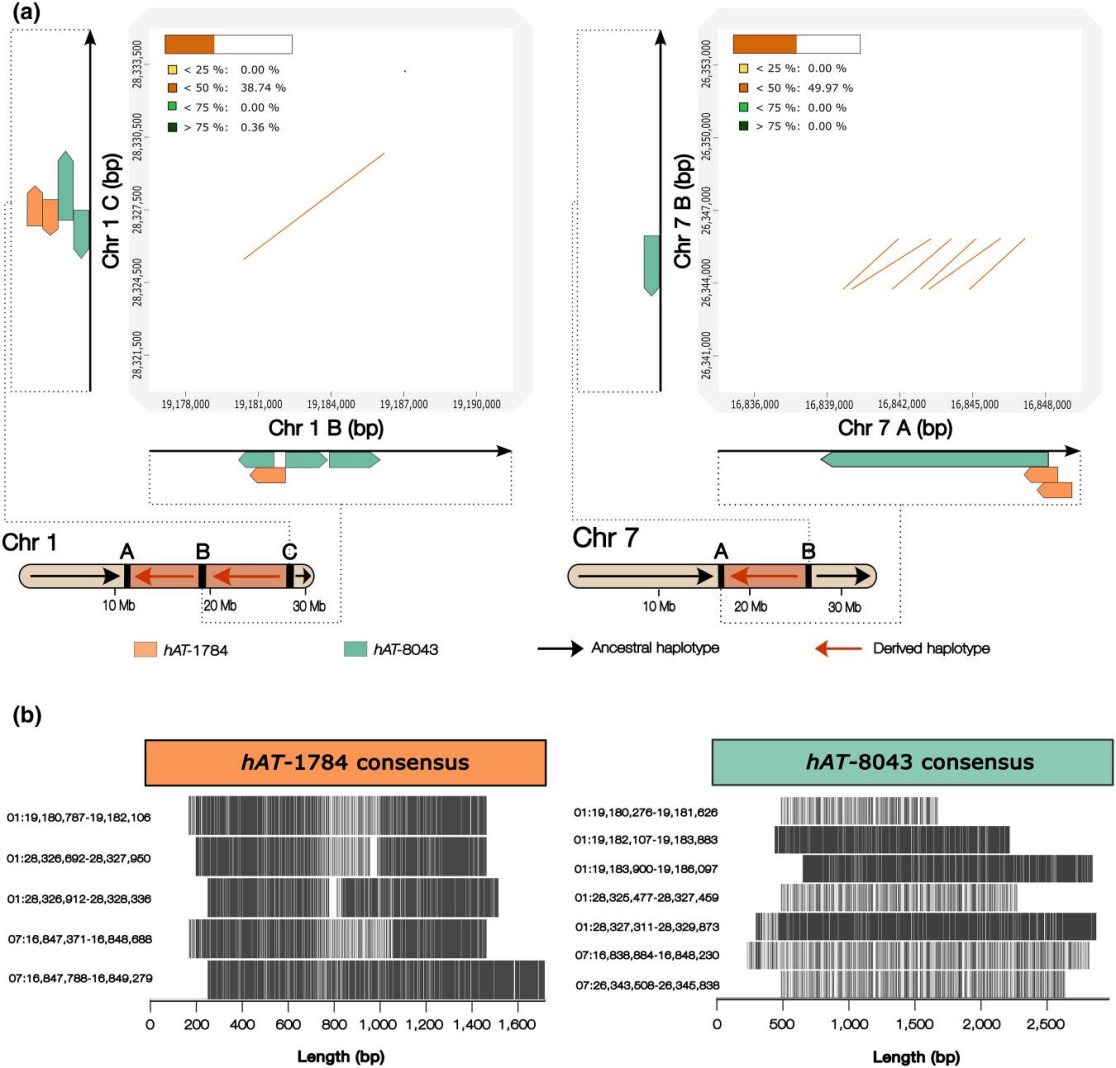
Accumulation of *hAT* TEs in the inversion breakpoints on chromosomes 1 and 7 in NEAC. a) Dot plot alignments of the breakpoint pairs in the derived (inverted) haplotypes on chromosome 1 (breakpoint B vs. C) and chromosome 7 (breakpoint A vs. B), highlighting sequence similarity between breakpoint regions (full alignments are shown in Fig. S11). Percentage identity of alignments is colored according to the inset and corresponds to regions with insertions from two *hAT* families, gadMor-*hAT*-1784 (orange) and gadMor-*hAT*-8043 (green). Insertions are drawn to scale, with strand orientations indicated on both axes. Red arrows denote the derived (inverted) haplotype. Chromosomes are drawn to scale, with sizes indicated in Mb. b) Mapping of the breakpoint insertions of gadMor-*hAT-*1784 and gadMor-*hAT-*8043 onto their respective consensus sequences, illustrating the portion of each consensus represented by the different copies. Sequence identity with the consensus is shown by black lines.

The derived breakpoints of chromosomes 1 and 7 harbored insertions of the same two particular families initially annotated as DNA *hAT* transposons. This is a common TE superfamily characterized by the presence of TIRs flanking an open reading frame (ORF) that encodes a transposase in their autonomous form (Atkinson 2015). These two breakpoint families were termed gadMor-*hAT*-1784 and gadMor-*hAT*-8043 (Figs. 3 and 4a). The gadMor-*hAT*-8043 insertions had a mean length of 1,973 bp (5 TEs; SD: 454 bp) and 5,838 bp (2 TEs) in chromosomes 1 and 7, respectively, whereas gadMor-*hAT*-1784 had a mean length of 1,334 bp (3 TEs; SD: 84 bp) in chromosome 1 and two insertions in the chromosome 7 breakpoint A (mean length 1,404 bp), overlapping an insertion of the gadMor-*hAT*-8043 family (Fig. 4a). In the syntenic breakpoint regions of the ancestral haplotype, gadMor-*hAT*-1784 and gadMor-*hAT*-8043 were present only in the syntenic breakpoint A in chromosome 1 (mean lengths: 1,385 and 890 bp for gadMor-*hAT*-1784 and gadMor-*hAT*-8043, respectively).

The insertions of gadMor-*hAT*-1784 elements were either overlapping the center of the breakpoints (e.g. breakpoint B and C in chromosome 1) or located in the immediate vicinity of the breakpoints (chromosome 7). Moreover, the breakpoint regions containing these *hAT* insertions exhibited a tandem repetitive pattern when aligned (Fig. 4a), and the inserted *hAT* copies represented the same part of the gadMor-*hAT*-1784 consensus sequence (Fig. 4a and b; Table S6). We observed the same patterns for the gadMor-*hAT*-8043 breakpoint insertions, which had overlapping insertions with several of the gadMor-*hAT*-1784 insertions (Fig. 4b).

**Fig. 5.**
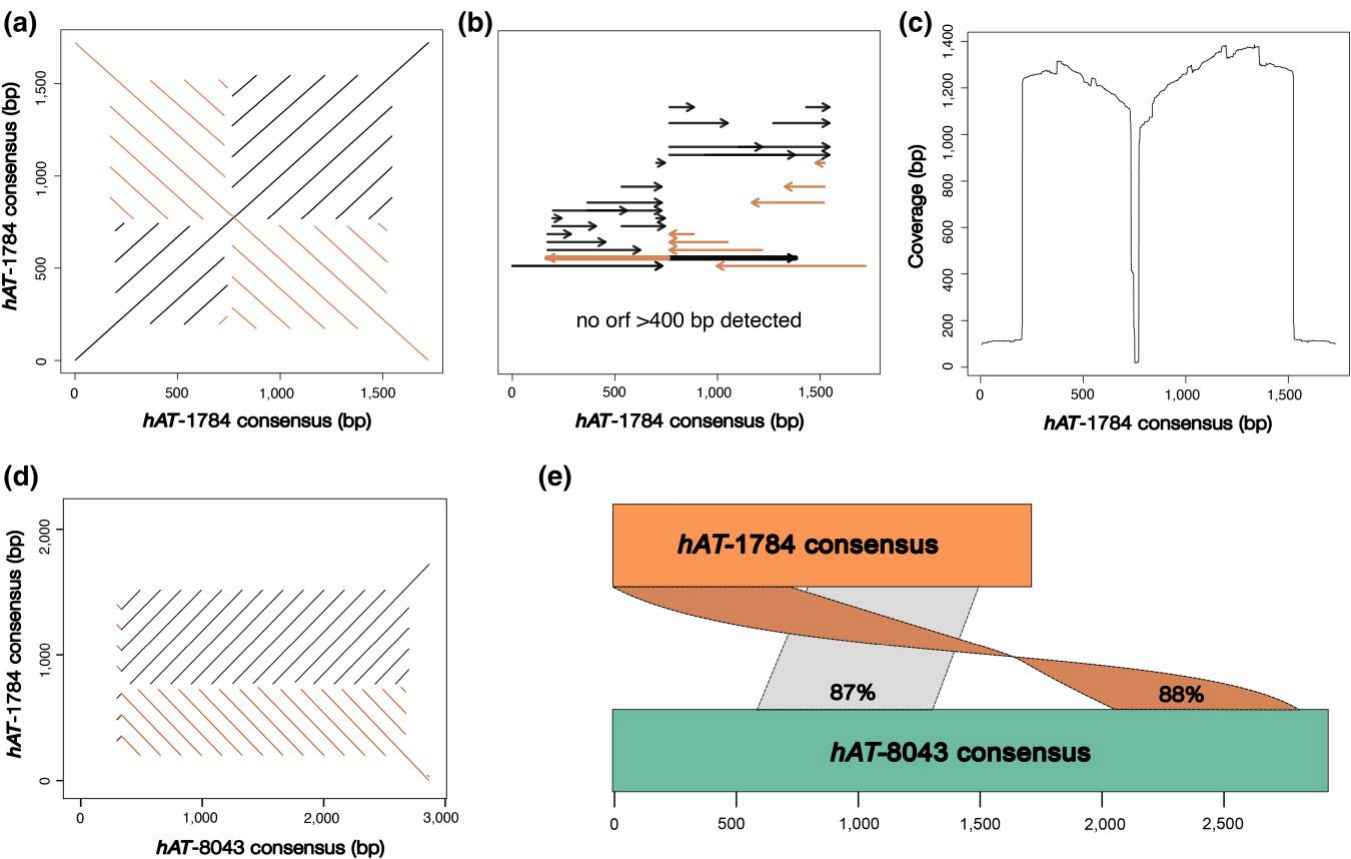
Structural characteristics of the *hAT*-derived MITEs in Atlantic cod. a) Self-alignment dot plot of the gadMor-*hAT*-1784 consensus sequence, showing the inverted repeat structure. b) Internal repeats of the gadMor-*hAT-*1784 consensus, with black arrows (rightward) indicating forward repeats and orange arrows (leftward) indicating inverted repeats. c) Genomic coverage of the gadMor-*hAT*-1784 consensus, showing the pileup of genomic hits across the consensus sequence. d) Dot plot alignment between the gadMor-*hAT*-1784 and gadMor-*hAT*-8043 consensus sequences, showing sequence similarity between the two TE families. e) Mapping of the gadMor-*hAT*-1784 (orange) onto the gadMor-*hAT*-8043 (green) consensus, with sequence identity for mapped regions indicated in percentages. Alignments in the forward direction are shown in gray, while reverse orientations are shown in orange.

To investigate the structural characteristics of gadMor-*hAT-*1784 on chromosomes 1 and 7, we utilized TE-Aid (Goubert et al. 2022). Self-alignment dot plots of the gadMor-*hAT-*1784 consensus sequence revealed a complex repeated sequence motif consisting of two large TIRs of ∼600 bp, which were made of multiple tandem repeats with repeating units of 167 to 169 bp (Fig. 5a and b). We did not identify any ORFs in the consensus sequence of gadMor-*hAT*-1784, and mapping of the consensus to the genome showed high genomic coverage for the long 600-bp TIRs, but not for the short internal sequence of the consensus (Fig. 5c). This indicates a near-complete loss of the internal transposase-encoding sequence of the gadMor-*hAT*-1784 TE. The presence of inverted repeats and internal deletions suggests that gadMor-*hAT-*1784 is a *hAT*-derived nonautonomous MITE with multiple internally inverted repeats in tandem (Fig. 5a and b).

We performed the same TE structural analysis on the gadMor-*hAT-*8043 consensus, which yielded a repetitive pattern resembling a simple sequence repeat with similar repeating units of ∼167 bp (Fig. S15). Since the gadMor-*hAT*-1784 and gadMor-*hAT*-8043 families shared a tendency to overlap the breakpoints in chromosome 1 and 7 and were often annotated in the vicinity of each other in a tandem arrangement of similar sized repeating units, we tested whether they share a common origin. We therefore compared the consensus sequences and structural characteristics of the two families following the 80-80-80 rule (Wicker et al. 2007) for families and 80-95-98 rule for subfamilies. This suggests that gadMor-*hAT*-1784 and gadMor-*hAT*-8043 represent two different nonautonomous subfamilies of the same ancestral *hAT* family (Fig. 5d and e).

**Fig. 6.**
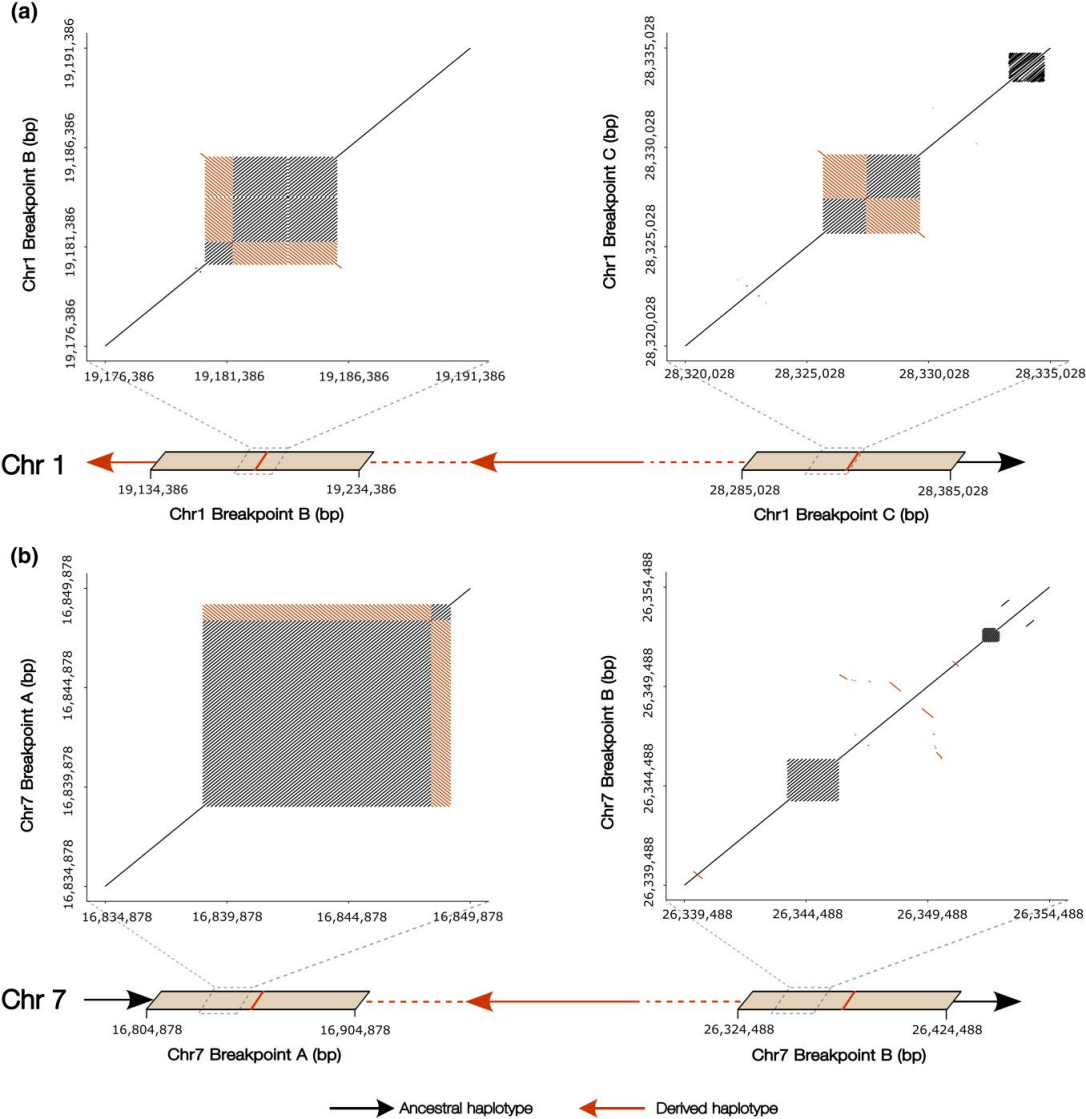
Self-alignment dot plots of breakpoint regions on chromosomes 1 and 7 containing gadMor-***hAT***-1784 and gadMor-***hAT***-8043 insertions. a) Chromosome 1 breakpoint self-alignment. b) Chromosome 7 breakpoint self-alignment. Red arrows (leftward) indicate an inversion, and black arrows (rightward) indicate regions outside the inversion. The red lines represent the midpoint coordinates of the breakpoint estimates from Table 1. Alignments in the forward direction are shown in gray, while reverse orientations are shown in orange.

We validated the repetitive structure of both *hAT* subfamilies by performing self-alignment dot plots of the breakpoint regions spanning the insertions of the gadMor-*hAT*-1784-derived MITE and gadMor-*hAT*-8043-derived simple sequence repeat on chromosomes 1 and 7, confirming the presence of long tandemly inserted MITE and simple sequence repeats within the breakpoints, which resemble the same parts of the gadMor-*hAT*-1784 and gadMor-*hAT*-8043 consensus sequences (Fig. 6).

### Inversion Breakpoints on Chromosome 2 Contain Heterogenous TE Family Landscapes

We identified insertions from several TE families within the breakpoints of the chromosome 2 inversion (Fig. 3). These families represented a diverse landscape of TE families, often with multiple insertions (2 to 5 per family) of varying length (131 to 1,757 bp), primarily belonging to the retroelements. Most of these TE insertions were shared between the two inversion haplotypes (i.e. derived and ancestral). However, two LTR retrotransposon families—gadMor-Ty3/Metaviridae-342 and gadMor-Ty3/Metaviridae-93—were specific to the derived breakpoint regions and absent from the corresponding ancestral regions.

To investigate the breakpoints in more detail and evaluate the TE insertion patterns across individuals (and not only in the reference genomes), we used long-range PCR and long-read sequencing (PacBio HiFi; see Materials and Methods) to amplify the chromosome 2 breakpoint regions. We successfully sequenced 39 individuals of NEAC and NCC from different locations (Table S7), including homozygous and heterozygous individuals for the two inversion haplotypes. By mapping reads from heterozygous individuals to the NEAC and NCC reference genomes (using coordinates from Table 1)—where NEAC carries the ancestral haplotype and NCC the derived haplotype—we were able to validate our bioinformatics-led estimations of the breakpoint coordinates and further refine the breakpoint intervals on chromosome 2 to ranges of 111 to 266 bp (Table S8 and Fig. S16).

One feature of these breakpoint sequences was their low sequence variation across the sequenced individuals (Fig. S1^6^). This pattern extended to the level of TE insertions, where one of the two LTR retrotransposon families—gadMor-Ty3/Metaviridae-342—was consistently found in the breakpoint regions of all individuals carrying the inverted haplotype. These insertions represented internal, protein-coding regions of the TE family (Table S6), and the insertions in breakpoint A were oriented in the reverse direction of the breakpoint B insertions. The regions harboring the remaining TE breakpoint families that were shared across the derived and ancestral haplotypes were not successfully amplified, as they were located outside the range of the sequenced regions.

## Discussion

The genomic signatures of various molecular mechanisms leading to chromosomal inversions are largely underexplored using genomic data. Here, we have identified and characterized the TE content in the breakpoint regions for the four chromosomal inversions previously identified in Atlantic cod (Berg et al. 2016, 2017; Matschiner et al. 2022). This involved obtaining a curated lineage-specific TE library, as suggested by best practices for proper characterization and annotation of repetitive DNA content (Platt et al. 2016; Peona et al. 2021a; Goubert et al. 2022; Storer et al. 2022). Our most notable finding is that certain TE orders (e.g. MITEs and Mavericks) and superfamilies (*hAT* elements) display elevated densities (i.e. the proportion of TE insertions per sliding window) within breakpoints relative to other areas of the genome. Specifically, we identified similar TE families in the breakpoint pairs of all four inversions. The shared pattern of family-specific TE accumulations in the derived inversion breakpoints indicates a clear association between TE insertions and inversion breakpoints (see scenario a in Fig. 1).

Adding to the observed accumulation of homologous TEs in the four breakpoints pairs, we found that the breakpoints of the derived haplotypes for chromosomes 1 and 7 harbored long TEs (>1,000 bp) of the same family, which we demonstrate is an unlikely association to detect by chance across the genome. When aligning the two breakpoint regions associated with each inversion, we found a higher sequence identity in regions that contained related TEs compared to adjacent regions. Furthermore, our amplification of the breakpoint regions on chromosome 2 in multiple cod individuals showed that the breakpoint insertions of TEs were conserved across the sequenced individuals, which can be extrapolated to the remaining three inversions in chromosomes 1, 7, and 12. Together, the observation of sequence similarity and preserved insertions of specific TE families seems to represent a general feature of the four major inversions in the Atlantic cod.

We observed tandemly repeated *hAT-*insertions within the inversion breakpoints of chromosomes 1 and 7. *hAT* elements are known to be abundant in teleost fish (Gao et al. 2016), and previous studies have suggested *hAT*s as drivers for creating inversions in fruit fly (*Drosophila melanogaster*) (Lyttle and Haymer 1992), maize (*Zea mays*) (Zhang and Peterson 2004; Sharma and Peterson 2023), yeast (*Saccharomyces cerevisiae*) (Sarilar et al. 2014), and structural variants observed in aphids (Mathers et al. 2021). We classified the breakpoint *hAT* elements in the Atlantic cod as MITEs because they lacked the transposase but still preserved the inverted repeat regions. This is particularly evident from the gadMor-*hAT*-1784 consensus sequence, which lacks a transposase, and is further exaggerated in the subfamily gadMor-*hAT*-8043, which has only preserved one of the TIRs in a tandem repeat arrangement. The complex, nested pattern of *hAT*-derived MITEs is unique to the breakpoint pairs of chromosomes 1 and 7, suggesting a causal link between these elements and the origin of the two inversions.

The presence of long inverted repeats likely facilitated the origin of inversions through ectopic recombination. Inversions that occur by NHEJ leave distinct genomic signatures that are independent of TE accumulations; e.g. inverted duplications of nonrepetitive DNA, as shown in Fig. 1 (Ranz et al. 2007; Furuta et al. 2011; Guillén and Ruiz 2012), whereas inversions formed by ectopic recombination are typically characterized by accumulations of related TEs and inverted, chimeric repeats (Delprat et al. 2009; Guillén and Ruiz 2012). We therefore distinguished these mechanisms by investigating the TE sequence similarity in the breakpoint and elucidating the shared structural characteristics (e.g. TIRs and LTRs) of their insertions. In line with an ectopic recombination scenario, the characterization of the breakpoint sequences revealed long stretches of similar inverted repeats caused by long MITE insertions. Indeed, MITEs are associated with inversions in various study systems, such as *Drosophila repleta* (Rius et al. 2013) and *Brassica napus* (Wan et al. 2017), and recombination between short inverted sequences of TEs has been shown to generate inversions in bacterial chromosomes (Ling and Cordaux 2010). Our results demonstrated accumulations of *hAT*-derived MITEs spanning several thousands of base pairs in the breakpoints of chromosomes 1 and 7 (mean lengths spanning 1,334 to 5,838 bp) with long repeating units (167 to 169 bp). We propose that the presence of these long inverted tandem repeats facilitated the inversions by ectopic recombination, This scenario is further supported by our manual inspection of the two *hAT*-consensus sequences (gadMor-*hAT*-1784 and gadMor-*hAT*-8043) and their breakpoint insertions, which confirmed that their insertions consistently represented the same or overlapping portions of their respective consensus sequences, leaving a genomic signature of chimeric MITEs that resembles the ectopic recombination scenario described in Fig. 1. Finally, the lack of clear signals of NHEJ in any of the inversion breakpoints implies that TEs have facilitated the four Atlantic cod inversions through recombination of similar TEs.

Our results for chromosomes 1, 7, and 12 revealed a distinct pattern where certain related TEs of type II DNA elements were only associated with the breakpoints of the derived haplotypes, while absent from the corresponding ancestral regions. Since inverted haplotypes typically experience reduced recombination rates (Wellenreuther and Bernatchez 2018; Villoutreix et al. 2021), this reduction can lead to local population structuring within the inverted regions, which are characterized by high linkage disequilibrium. Such disequilibrium often extends beyond the inversion breakpoints (Matschiner et al. 2022). As a result, these regions can act as “low-recombination refugia,” where an accumulation of TEs is expected (Peona et al. 2021b), effectively sheltering the TE insertions from degrading. Thus, TEs could have accumulated in the Atlantic cod breakpoints following the inversion due to low recombination. Given this scenario, we would expect accumulations of various TEs (not necessarily similar families) across the entire inverted region. However, our findings show that TE accumulation is concentrated within the breakpoint regions, with no evidence of elevated TE densities in the rest of the inversion. Moreover, the insertions within breakpoints tend to be of the same families, where the insertions overlap similar regions of their respective TE consensus sequences. This argues for related TE accumulation in the breakpoint pairs due to an association with the inversion origin. In contrast, the ancestral haplotype would likely have experienced normal recombination rates following the inversion origin, allowing TEs to transpose or decay, which provides a potential explanation for the absence of related TEs in the ancestral syntenic breakpoints on chromosomes 1, 7, and 12.

In contrast, our analysis of the chromosome 2 breakpoints revealed a predominance of retroelement insertions, many of which were present in both the ancestral and derived breakpoints. Chromosomal rearrangements in certain avian genomes (Boman et al. 2019) and yeast (*S. cerevisiae*) (Chan and Kolodner 2011) show a positive correlation with enrichments of retroelements—particularly the LTR retrotransposons—which have generated chromosomal inversions in the fruit fly *D. melanogaster* by mediating ectopic recombination (Lim and Simmons 1994). Unlike DNA elements, retroelements transpose via reverse transcription, which leaves behind copies at their original genomic locations. We observed numerous related TEs in the breakpoint pairs of both the ancestral and derived haplotypes in chromosome 2, but no elevation of general TE density. The shared TE families between the ancestral and derived inversion haplotypes on chromosome 2 could indicate that these TEs have been retained in both arrangements. However, in the case of the HiFi-sequenced breakpoints on chromosome 2, we only sequenced regions harboring one of the LTR retrotransposon families that were associated with the derived and not the ancestral breakpoint regions (i.e. gadMor-Ty3/Metaviridae-342). Common for these breakpoint insertions was their location at the outer edges of the two breakpoint regions, with the different breakpoints harboring insertions in the opposite orientation of one another. Each breakpoint insertion represented internal parts (ORFs) of their consensus sequences. Furthermore, the insertions showed substantial divergence from their consensus sequences, with a mean divergence of 31.1% for the gadMor-Ty3/Metaviridae-342 insertions. The high divergence suggests that the breakpoint insertions represent old, nonautonomous TE copies that may have been preserved at these sites since before the origin of the inversion, which is estimated at 0.88 Ma (Matschiner et al. 2022).

The identification of TE-derived tandem repeats within the various inversion breakpoints adds to recent discoveries of the impact of tandem-repeated TEs in shaping genomes. This is exemplified in the 9 kb tandemly inserted TE influencing melanism in the peppered moth (Hof et al. 2016). Inserted TEs can elongate by unequal crossover and replication slippage, or they can be inserted in tandem configurations (McGurk and Barbash 2018). Indeed, DNA class II transposons, such as the ones found here in the inversion breakpoints, commonly perform double insertions in a head-to-tail orientation (McGurk and Barbash 2018), making them susceptible for ectopic recombination and subsequent structural alterations, such as inversions. Given the extensive TE diversity and particular enrichment of class II TEs in teleost fish (Gao et al. 2016; Reinar et al. 2023), along with the prevalence of inversions (Natri et al. 2019; Matschiner et al. 2022; Andersson et al. 2023; MacGuigan et al. 2023) and other structural variants documented in cod and other teleosts, e.g. loss of MHC II in cod (Star et al. 2011; Malmstrøm et al. 2016), loss of MHC II in pipefishes (Haase et al. 2013; Small et al. 2016; Roth et al. 2020), loss of MHC II in anglerfish species (Dubin et al. 2019; Swann et al. 2020), hemoglobin MN cluster duplications in nine-spined stickleback and codfishes (Baalsrud et al. 2017; Varadharajan et al. 2019), and copy number variations of antifreeze glycoprotein-encoding genes in codfishes and notothenioids (Baalsrud et al. 2018), it becomes essential to thoroughly characterize and quantify TEs as potential evolutionary drivers of structural variants in general. Only then will we be able to obtain a better understanding of the drivers of the origin of structural variants. Our study highlights the propensity of certain TE structural features, such as TIRs, to promote inversions when present in tandem arrangements. In contrast to inversions originating from NHEJ, all of the four large inversions in Atlantic cod likely originated through ectopic recombination involving TEs, both DNA elements and retroelements.

## Materials and Methods

### Data

We used three high-quality reference assemblies for this work: the NEAC assembly (NCBI accession ID: GCF_902167405.1; contig N50 = 1,015 kb, scaffold N50 = 29 Mb, and BUSCO score = 96.8%), the NCC genome assembly (NCBI accession ID: GCA_964260575; contig N50 = 271 kb, scaffold N50 = 28 Mb, and BUSCO score = 90.5%), which are both based on PacBio long-read sequencing and chromosome-resolved with Hi-C linked reads, and the Oxford Nanopore-based Celtic cod assembly (NCBI accession ID: GCA_010882105.1; contig N50 = 10,560 kb, scaffold N50 = 27 Mb, and BUSCO score = 94.1%). Detailed description on sequencing and assembly construction of the three assemblies can be found in Jentoft et al. (2024), Hoff et al. (2024), and Kirubakaran et al. (2020), respectively.

### Estimating the Inversion Breakpoint Coordinates

To determine the location of the breakpoint regions for the inversions located on chromosomes 1, 2, 7, and 12, we aligned the reference genome for NCC to the reference genome of NEAC using minimap v.2.26 (Li, 2018) and SyRi (Goel et al. 2019). This allowed the identification of syntenic regions and structural variants between the two reference genomes, including inversions. As a complementary analysis, we dot plotted chromosomes 1, 2, 7, and 12 from NEAC against their corresponding homologous chromosomes in NCC to visually inspect the presence of inversions with D-GENIES v.1.4 (Cabanettes and Klopp 2018). The breakpoint regions were not identified as exact locations on either genome, but rather as approximate regions in which the breakpoints were located (Table S1a and Figs. S1 and S2), surrounded by clear homologous regions between the two genomes, one of which was in opposite direction to the other. We cross-referenced the breakpoint coordinates with the inversion configurations and breakpoints present in the Celtic cod assembly, which is assumed to have inversion haplotypes that are similarly oriented as the NCC (Kirubakaran et al. 2020). Our synteny-based identification of breakpoints indicated that the chromosome 7 and 12 inversions might be double inversions, the former with overlapping breakpoints. Since we do not have population data to support this (nor do any other previous contributions on the cod inversions clearly suggest this), we are treating them as single inversions (breakpoint locations highlighted in Fig. S2).

To obtain more precise estimations of the breakpoint regions, we mapped raw reads from the NEAC and NCC assemblies back onto both of the assemblies using minimap v.2.26 (Li, 2018). When aligning reads back to their original assembly (e.g. NEAC reads to the NEAC reference genome), reads that spanned an inversion breakpoint mapped continuously, without interruption. In contrast, when reads were aligned to the alternative assembly (e.g. NEAC reads to the NCC reference genome), those spanning the inversion breakpoints would only partially map to one boundary, with the remaining portion aligning in reverse orientation to the opposite boundary. We evaluated read coverage around putative breakpoint regions using pyGenomeTracks (Lopez-Delisle et al. 2021) (Fig. S3), where we assumed that there would be a lower coverage around breakpoint regions from cross-mapped reads (e.g. from NCC onto NEAC) than from mapping reads from the same assembly (e.g. NEAC onto NEAC). Finally, we visually inspected hard-clipped reads (i.e. reads where a portion has not aligned well to the reference genome well and is thus excluded from the alignment) as labeled by minimap 2.26, using GenomeRibbon (Nattestad et al. 2021). The hard-clipped reads that mapped to two locations in opposite directions (i.e. mapped to two breakpoints) marked the boundaries of the inversion breakpoint intervals (Fig. S4). To infer syntenic breakpoint regions in the ancestral (noninverted) haplotypes, we used the estimated breakpoint intervals and adjacent regions (midpoint of breakpoint interval ± 50,000 bp) and mapped these regions onto the assembly carrying the ancestral haplotype (for each inversion), using minimap v.2.26. The outer boundaries of the mapped regions defined the edges of the syntenic breakpoint regions (Table S1b).

### HiFi-Sequencing and Population-Level Variant Calling of Chromosome 2 Breakpoints

We obtained 58 tissue samples from two wild populations of Atlantic cod, Lofoten (*N* = 30) and Averøya (*N* = 28). Additionally, we obtained three individuals from the Atlantic cod breeding program for aquaculture at Nofima, Tromsø, for establishing and optimizing the PCR protocol for sequencing the inverted and noninverted breakpoint region in chromosome 2. DNA was extracted from 0.025-g tissue samples using the DNeasy Blood and tissue mini kit by QIAGEN. All samples were extracted according to the manufacturer's instructions in “DNeasy Blood & Tissue Handbook, July 2020.” The concentration and purity of the DNA were estimated using NanoDrop (Thermo Fisher Scientific) and a Qubit fluorometer (Invitrogen, Thermo Fisher Scientific).

Library preparations for PacBio HiFi sequencing were done by the Norwegian Sequencing Center on ∼25% of 8M SMRT cells using Sequel II Binding kit 2.0 and Sequencing chemistry v2.0. The sequencing data were demultiplexed with the demultiplexing pipeline on SMRT Link v10.2.0.1333434, and circular consensus sequencing (CCS) reads were then generated

To analyze breakpoint variation in chromosome 2, we mapped sequenced against NEAC and NCC reference genomes using minimap2 (Li 2018). We were able to map a total of 158 CCS sequences, and we used SAMtools (Danecek et al. 2021) to examine the mapped output bam files and BCFtools mpileup to call variants (Danecek et al. 2021) for NEAC and NCC. The resulting VCF files were used for haplotype phasing and tagging with WhatsHap v1.4 (Martin et al. 2016). These haplotypes were then assembled using Flye v2.9 (Kolmogorov et al. 2020). We then created de novo haplotype assemblies that we aligned together in a multiple sequence alignment using Geneious Prime 2021.2 (https://www.geneious.com) (Fig. S16).

To determine the breakpoint regions on chromosome 2 and their sequence variation, we compared the breakpoint sequences of each individual to bioinformatically determined breakpoint regions on the NEAC and NCC genome assemblies (Table 1). We focused on heterozygous individuals and compared the derived (inverted) breakpoint A to the syntenic breakpoint A region of the ancestral (noninverted) haplotype (Fig. S16) and performed the comparison for the breakpoint B haplotypes (Fig. S16).

### Repeat Annotation and Classification

To capture species-specific TE composition in Atlantic cod, we started by producing a de novo repeat library with RepeatModeler2 (Flynn et al. 2020). We ran RepeatModeler v2.0.1 on the NEAC assembly and included the built-in LTR structural discovery pipeline (Ellinghaus et al. 2008; Ou et al. 2018) to increase LTR detection. We then used MCHelper (Orozco-Arias et al. 2024) for automatic curation of our TE library, which involves identifying and removing false positives (i.e. multicopy genes and short tandem repeats), redundancy reduction, TE consensus elongation, structural checking using TE-Aid, and TE classification as suggested by best practices (see Goubert et al. 2022). MCHelper was run with the fully automated setting, with simple sequence repeat detection set to 90% and consensus Blast, Extract, and Extension cycle set to 8 rounds in MCHelper. We then focused on the nonredundant, unclassified TEs for manual processing and curating TEs. An overview of the pipeline we constructed for manual curation of TEs can be found in Fig. S5. In short, the pipeline consists of four steps: (i) TE classification of the nonredundant library using RepeatClassifier v.2.0.4 (only the Dfam 3.8 curated database) and PASTEC (Hoede et al. 2014); (ii) generation of a priority list for manual curation by masking the NEAC assembly with the unclassified, nonredundant TE library using RepeatMasker v.4.1.5 (Smit et al. 2013). Priority was based on insertions in the vicinity of inversion breakpoints and general abundance in the genome; (iii) manual curation of prioritized TEs using the output from PASTEC (structural and homology-based evidence), RepeatClassifier (built-in homology-based tool in RepeatModeler2), MCHelper, and manually inspection of the output from TE-Aid and multiple sequence alignments of TE consensus aligned to the assembly. Finally, we performed (iv) automatic curation of the remaining uncurated library using RMBlast v.2.13.0 and the curated TEs as a database. This outputted a final curated, classified TE library that was used to mask the NEAC, NCC, and Celtic cod assemblies using RepeatMasker v.4.1.5 with slow search for high sensitivity searches (see library comparisons in Fig. S17). To evaluate the quality of the final TE library, we compared the length and contiguity of the TE annotations of the raw versus the final curated library using the NTE50 and LTE50 metrics, as suggested by Jamilloux et al. (2016). NTE50 represents the number of largest TE copies required to annotate 50% of repetitive DNA, while LTE50 refers to the length threshold at which 50% of the repetitive DNA is annotated by copies longer than this length. Furthermore, the final TE library contained an overall longer and fewer consensus sequences (Fig. S17), indicating less false positives, lower consensus redundancy, and more complete consensus sequences.

To determine the population-level frequencies of TEs in breakpoints, we used the HiFi reads of the inversion in chromosome 2 and examined whether these reads overlapped with positions of annotated TEs using a genome browser (IGV v.2.12.3). We also used the Atlantic cod species-specific TE library to mask individual reads to confirm each observed breakpoint TE.

### Aligning Inversion Breakpoints to Investigate the Presence of Related TEs

To determine the repeat landscape of the different breakpoints and better capture the TE content in the regions flanking a breakpoint in a broader genomic context, we extended each breakpoint interval (of the derived inversion haplotypes) by taking the interval midpoints and expanding the regions by 50 kb in both directions. This resulted in equally sized breakpoint regions of 100 kb.

We evaluated the sequence similarity between breakpoint pairs of the four inversions by dot plotting the breakpoint region pairs associated with each inversion using D-GENIES v.1.4 (Cabanettes and Klopp 2018). If TEs are associated with an inversion, in concordance with a scenario of ectopic recombination, we expect the breakpoints to align due to similarity of repeats. We also expect to find related TE copies within the aligned regions. To ensure we limited our investigation to putative homologous sequences, only alignments with sequence identity exceeding 25% were retained and screened for related TEs. TE fragments that were less than 100 bp in length, and thus considered less likely to facilitate ectopic recombination, were excluded, and the remaining TEs were grouped by family. The TE families that were present in breakpoint pairs were manually investigated by curation of the consensus with structural and homology-based evidence from PASTEC (Hoede et al. 2014), MCHelper (Orozco-Arias et al. 2024), and manual searches in the Dfam (Storer et al. 2021) and Censor (Kohany et al. 2006) databases and by evaluating the insertions of copies to evaluate the number and length of the insertions, mean divergence from the respective consensus sequences, and whether the insertions overlapped the same parts of the consensus.

### Structural Validation of the gadMor-hAT-1784-Derived MITE

We used TE-Aid to validate structural features of MITEs associated with gadMor-*hAT-*1784 that appeared in multiple inversions (Goubert et al. 2022). Given the presence of numerous internally inverted repeats within the consensus sequence of gadMor-*hAT-*1784, we extracted the breakpoint regions from chromosomes 1 and 7 containing gadMor-*hAT-*1784 insertions and used self dot plots of each breakpoint to visualize palindromic insertions. To obtain this dot plot, we used BLASTn v.2.6.0+ (Altschul et al. 1990; Camacho et al. 2009) to compare the breakpoint insertions to the palindromic gadMor-*hAT-*1784 consensus.

We aligned the gadMor-*hAT*-1784 consensus sequence with the overlapping gadMor-Unc-8043 consensus with MAFFT v.7.520 (L-INS-i) (Katoh and Standley 2013)to investigate sequence similarity. To further evaluate if the two consensus sequences were part of the same family and/or subfamilies, we used CD-HIT v.4.8.1 (Fu et al. 2012) and applied the 80-80-80 rule for family identification (i.e. family members are at least 80 nucleotides in length and share 80% of residues over at least 80% of their length) and the more stringent 80-95-98 rule for subfamily identification. This showed that gadMor-Unc-8043 and gadMor-*hAT*-1784 are two subfamilies of the same family, and the two consensus sequences were grouped together as the same *hAT* family (gadMor-*hAT*-1784) for further analyses.

Additionally, to confirm the tandem-repeated MITE-structure inherent in the gadMor-*hAT-*1784 insertions, we employed ULTRA (Olson and Wheeler 2018) to identify and annotate tandem repeats in the NEAC assembly. ULTRA v.0.99.17 was run with a maximal detectable repeat period of 1,000 bp, and we checked whether the annotated tandem repeats overlapped with the gadMor-*hAT-*1784 and gadMor-*hAT*-8043 insertions in the chromosome 1 and 7 inversions in NEAC using a genome browser (IGV v.2.12.3).

### Estimating Putative Centromeres

To obtain statistical inferences of the density of TEs in breakpoint regions (see section Statistical Assessment of TE Features Within Breakpoints below), we needed to account for centromeric regions, as these regions are composed of high-density repeats, potentially biasing our inferences. We predicted putative centromeric regions in silico using three independent approaches. First, we plotted the density of interspersed repeats (from our TE library) and tandem repeats (as annotated by the TRF module included in RepeatMasker) on chromosomes 1, 2, 7, and 12, using nonoverlapping sliding windows of 10 kb. Second, we downloaded the putative 158-bp centromeric repeat sequence that is suggested to represent the centromeric repeat in Celtic cod (Kirubakaran et al. 2020) and mapped the sequence onto chromosomes 1, 2, 7, and 12 in the NEAC and NCC assemblies using BLAST v.2.14.1 (Altschul et al. 1990; Camacho et al. 2009). We compared the density peaks of interspersed and tandem repeats with the locations of the Celtic cod centromere sequence and used the corresponding density peaks for further processing. Third, we used CentroMiner v.1.2.1 (Lin et al. 2023) to predict centromere candidates on each of the four chromosomes and compared the output to the general repeat density peaks and the Celtic cod centromere sequence. Finally, we extracted the regions containing candidate centromeric regions and self-aligned these regions to identify potential repetitive motifs (e.g. simple repeats and palindromes), characteristic of centromeres. This holistic approach provided a final set of coordinates for the putative centromeres in these four chromosomes, which were excluded in further statistical analyses.

### Statistical Assessment of TE Features Within Breakpoints

We conducted two distinct statistical analyses on all inversion breakpoints to determine TE density and the probability of finding similar TE copies in breakpoint pairs. First, we plotted the mean TE density across chromosomes 1, 2, 7, and 12 in NEAC and NCC using nonoverlapping sliding windows of 25, 50, and 100 kb. We present the results using the 50-kb windows, since it was coherent with the size of the flanking sides of the breakpoint regions. To test whether the TE density in the breakpoints was significantly different from the chromosomal means, we did a Student's *t*-test for each chromosome, excluding centromere regions as described above. We repeated this analysis at the level of TE orders and superfamilies. We used a *t*-test for comparison because this test compares means and not median values, which would introduce bias in the results due to the density estimates being skewed toward zero in many windows, especially at the lower taxonomic levels of TEs. Additionally, we checked if the *t*-tests were biased due to the priority of TEs in the curation process (Fig. S18), by applying the same test to the raw, uncurated TE library. This showed a higher detection of MITEs after curation, but our results were unaffected at the level of superfamilies (e.g. the high levels of *hAT* elements in chromosome 7) (Fig. S18).

Second, we assessed the probability of encountering copies of the same TE family within both breakpoints of an inversion. For chromosomes where we found inversions (1, 2, 7, and 12), we generated 1,000 randomly selected region pairs (mock breakpoints) along the chromosome, with each region being 10 kb in size. We then calculated the average count of related TEs (from the same family) in the mock breakpoints, using different minimum TE insertion lengths: 0, 100, 500, 1,000, and 2,000 bp. This allowed us to estimate the probability of encountering longer TE insertions from the same family in randomly selected region pairs. We repeated this analysis for increasing region sizes, ranging from 10 to 100 kb in 10-kb increments. The average counts of related TEs in the mock breakpoints were then compared to the observed counts of related TEs in the real inversion breakpoints.

### Evaluating the Signatures of Ectopic Recombination and NHEJ in Inversion Breakpoints

To evaluate the molecular processes most likely involved in the origin of the four different cod inversions, we assessed the breakpoint signatures of NHEJ and ectopic recombination, as illustrated in Fig. 1. We used the structural variants outputted from SyRi (Goel et al. 2019; see Estimating the Inversion Breakpoint Coordinates) to identify inverted duplications in the breakpoint regions (midpoint ± 50 kb) of the derived haplotypes (Fig. S14). Since inverted duplications in the case of NHEJ are usually not linked to TEs, we double-checked if any of the inverted duplications were annotated as TEs in opposite directions, which would imply ectopic recombination. If the inverted duplications in a breakpoint were not annotated as TEs and corresponded to a duplicated region in the syntenic breakpoint of the ancestral haplotype, we interpreted this as evidence for NHEJ, where the segment in the ancestral breakpoint has been duplicated in opposite direction during DNA repair following strand breakage (Furuta et al. 2011; Burssed et al. 2022). The process of evaluating inversion formation by ectopic recombination of TEs has been described in detail above. If we observed molecular signatures of both processes, i.e. same-family chimeric TEs and non-TE inverted duplications, we assessed the distance from the insertions to the boundaries of the breakpoint ranges presented in Table 1. The molecular signatures that were positioned closest to the breakpoints were deemed the most likely molecular mechanism in generating the inversion.

## Supplementary Material

evaf131_Supplementary_Data

## Data Availability

Data are available in the Figshare database (PacBio HiFi reads from cod individuals are https://doi.org/10.6084/m9.figshare.24339823.v1).
